# The Erlangen test of activities of daily living in persons with mild dementia or mild cognitive impairment (ETAM) – an extended validation

**DOI:** 10.1186/s12888-018-1886-5

**Published:** 2018-09-24

**Authors:** Stephanie Book, Katharina Luttenberger, Mark Stemmler, Sebastian Meyer, Elmar Graessel

**Affiliations:** 1 Center for Health Services Research in Medicine, Department of Psychiatry and Psychotherapy, Friedrich-Alexander Universität Erlangen-Nürnberg, Schwabachanlage 6, 91054 Erlangen, Germany; 2 Institute of Psychology, Friedrich-Alexander-Universität Erlangen-Nürnberg, Nägelsbachstr. 49c, 91052 Erlangen, Germany; 3 Institute of Medical Informatics, Biometry, and Epidemiology, Friedrich-Alexander-Universität Erlangen-Nürnberg, Waldstraße 6, 91054 Erlangen, Germany

**Keywords:** Activities of daily living, Cognitive impairment, Dementia, Performance test, Validation

## Abstract

**Background:**

The ability to perform activities of daily living (ADLs) is a central marker in the diagnosis and progression of the dementia syndrome. ADLs can be identified as basic ADLs (BADLs), which are fairly easy to perform, or instrumental ADLs (IADLs), which involve more complex activities. Presently, the only performance-based assessment of IADL capabilities in persons with cognitive impairment is the Erlangen Test of Activities of Daily Living in Persons with Mild Dementia or Mild Cognitive Impairment (ETAM). The aim of the present study was to revalidate the ETAM in persons with mild cognitive impairment (MCI) or mild dementia and to analyze its application to persons with moderate dementia.

**Methods:**

We used baseline data from a cluster randomized controlled trial involving a sample of 443 users of 34 day-care centers in Germany. We analyzed groups of persons with MCI, mild dementia, and moderate dementia, categorized on the basis of the Mini-Mental State Examination (MMSE) and the Montreal Cognitive Assessment (MoCA). An item analysis was performed, and new discriminant validities were calculated. We computed a confirmatory factor analysis (CFA) to examine the postulated theoretical model of the ETAM with all six items loading on a single IADL factor. This was the first time that the ETAM’s sensitivity to change was analyzed after a time period of 6 months.

**Results:**

The overall sample scored on average 17.3 points (SD = 7.2) on the ETAM (range: 0–30 points). Persons with MCI scored on average 23.2 points, persons with mild dementia scored 18.4 points, and persons with moderate dementia scored 12.9 points, *p* < .001 (ANOVA). The item analysis yielded good difficulty indices and discrimination powers. The CFA indicated a good fit between the model and the observed data. After 6 months, both the ETAM score at baseline and the change in MMSE score (t0-t1) were significant predictors of the ETAM score at t1.

**Conclusions:**

The ETAM is a valid and reliable instrument for assessing IADL capabilities in persons with MCI or mild dementia. It is sensitive to changes in cognitive abilities. The test parameters confirm its application to persons with moderate dementia.

**Trial registration:**

Identifier: ISRCTN16412551 (Registration date: 30 July 2014, registered retrospectively).

## Background

The mastery of everyday practical capacities is essential for the elderly to maintain their independence. Lawton and Brody [1] defined a set of everyday activities for the elderly, so-called activities of daily living (ADLs). They differentiated between basic ADLs (BADLs), which refer to self-maintenance skills such as feeding, dressing, and toileting, and instrumental ADLs (IADLs), which cover more complex behaviors of domestic functioning and enable independent living. IADLs include food/meal preparation, financial administration, housekeeping, laundry, use of the telephone, responsibility for one’s own medication, mode of transportation, and shopping.

With an aging population, the number of people with dementia has dramatically increased in recent decades, and dementia has become a public health challenge [2]. Alzheimer’s disease is the most common form of dementia and begins years before the onset of clinical symptoms. Its pathology can be described on a continuum that ranges from a preclinical stage (changes in biomarkers) to a prodromal stage with minor cognitive symptoms/mild cognitive impairment, to a symptomatic stage that includes dementia [3, 4]. At different stages of the disease, different assessments are needed. While a patient is in the preclinical stage, an assessment of biomarkers is most important, whereas functional assessments become more important in the prodromal and symptomatic stages [5]. IADLs can be used for a functional assessment as early as in the prodromal stage because it has been shown that impairments in IADLs are associated with the diagnosis and development of dementia [6–10] and, more important, deficits in BADLs and IADLs seem to occur at different stages of the dementing process [10, 11]. Whereas BADLs have been found to be more strongly correlated with motor functioning and coordination [12] and thus are more likely to remain preserved until the later stages of the disease, IADLs have been found to be more sensitive to the earlier stages of cognitive decline as these activities are more complex and require greater neuropsychological organization [11]. Even more, IADL impairments have been shown to predict the progression to dementia and can be used to help distinguish between dementia and early forms of cognitive decline, such as mild cognitive impairment (MCI) [6, 13]. MCI refers to a state that is defined by the presence of the first cognitive impairments that do not yet constitute dementia [14] but have a high probability of progressing to dementia [15]. Persons with MCI can experience subtle changes in everyday functional competence [8]. There is scientific evidence showing that IADLs can be impaired in MCI [8, 16–18]. In addition, in a systematic review, Jekel et al. [17] reported that patients with MCI and IADL deficits seem to have a higher risk of developing dementia than patients with MCI without IADL deficits, again stressing the importance of IADLs.

Because there is ample evidence that the ability to perform IADLs plays a crucial role in identifying the development of the dementia syndrome, there is a need for assessment tools that have been specifically designed and validated for patients with the first signs of impairments in IADLs (i.e. persons with MCI or mild dementia). As one study showed that several informant-based IADL questionnaires were limited in their quality [19], it remains important to identify an optimal way to measure IADLs. A promising approach is the use of performance tests as these tests provide standardized and more objective results [17]. To move in this conceptual direction, the Erlangen Test for Activities of Daily Living (E-ADL) [20] was developed in 2009 and can be characterized by its excellent economy. In contrast to other performance tests, it requires only about 10 min to be performed and does not require any tasks to be done outside the test room. The E-ADL was designed to assess BADL capabilities and can be used with persons with moderate or severe dementia [21]. Because it is too easy for persons with less severe dementia, there is a need for a performance test that has been validated for persons with mild dementia or even MCI. For this reason, the Erlangen Test of Activities of Daily Living in Persons with Mild Dementia or Mild Cognitive Impairment (ETAM) was developed as a performance-based tool for the assessment of IADLs [22]. The ETAM addresses some of the disadvantages of existing performance tests for ADL capabilities as some of these are very time-consuming (from 45 min, Functional Living Skills Assessment [FLSA] [23], up to 1.5 h, Direct Assessment of Functional Abilities [DAFA] [24]), cover only a limited range of relevant domains of IADLs, or include culture-specific items (e.g. “calling directory assistance” or “refilling a prescription” in the Revised Direct Assessment of Functional Status [DAFS-R] [25]). Above all, the ETAM can be used with persons with MCI [22]. In a first validation study of 107 study participants, including participants with normal cognition, persons with MCI, and persons with mild dementia, the ETAM was shown to be a feasible performance-based assessment tool with good psychometric parameters [22]. In this first study, the final structure of the ETAM was developed, and the items were reduced from ten to six items on the basis of an exploratory factor analysis and other criteria.

However, because this study was only cross-sectional, there is currently no longitudinal data on the ETAM’s sensitivity to change. This is essential because sensitivity to change or responsiveness is an essential aspect of validity. It provides important information about the ETAM’s ability to measure change over time, and consequently, it determines whether the ETAM can be used in intervention studies. At this time, there are currently no performance tests for assessing IADLs in persons with MCI that can be used in intervention studies. Thus, one aim of the present study was to analyze the ETAM’s sensitivity to change. In addition, we wanted to investigate whether the original target group of persons with MCI or mild dementia could also be extended to include persons with moderate dementia. This would extend the application of the ETAM enormously because dementia is a progressive disease. Other aims of the present study involve other test construction criteria. The exploratory factor analysis in the validation study supported a one-factor structure for the ETAM. In the current study, we conducted a confirmatory factor analysis and investigated whether this structure could be supported. This was important to do in order to determine whether actual data were consistent with the hypothesis that the ETAM consists of a single IADL factor. Other test construction criteria included analyzing discriminant validity with additional instruments and determining criterion-related validity.

## Methods

### Design

**Fig. 1:**
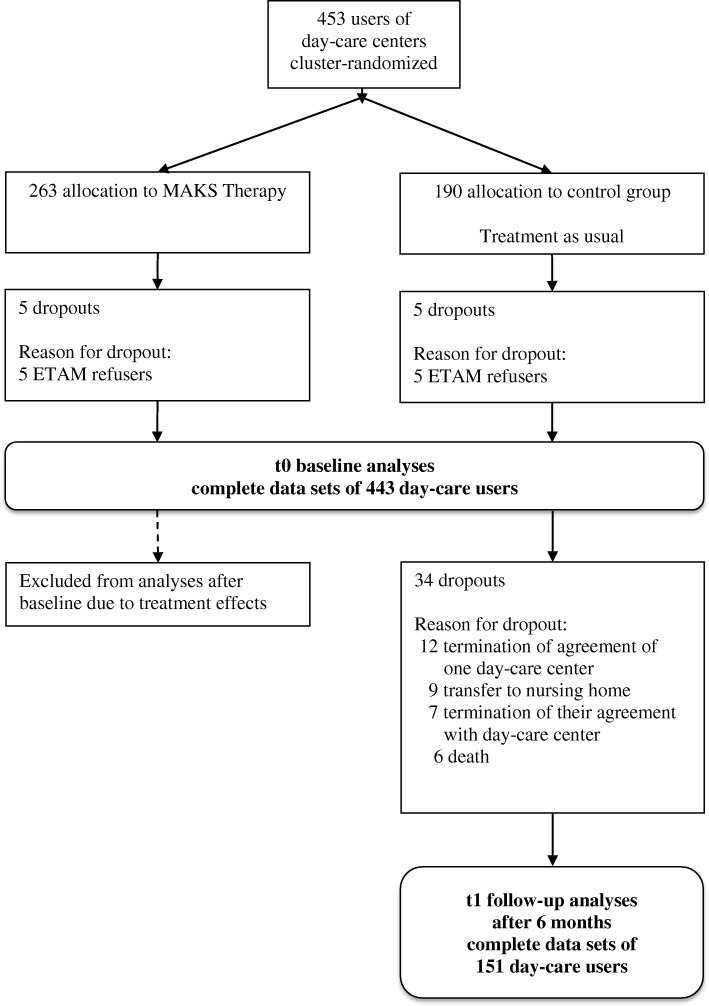
Consort flow chart

The data for the extended validation were obtained from the two-arm cluster randomized controlled trial “DeTaMAKS project” (ISRCTN16412551) to evaluate a six-month-long multimodal non-pharmacological therapy (MAKS therapy) in day-care centers in Germany with day-care-center users and their caregivers. The study protocol was published previously [26]. For the current study, we included baseline data (t0) from all day-care-center users and follow-up data after six months (t1) for the control group that received no study-specific treatment (Fig. 1). The MAKS intervention is a multimodal nonpharmacological therapy for older adults with mild to moderate dementia and has been shown to be an effective treatment for dementia [27]. Because of the influence of the MAKS therapy on the ETAM scores [28], all analyses with t1 data were computed only on data from the control group, which did not receive any special therapy during that time. Cross-sectional analyses from the first measurement point (t0) were computed on data from all participants (the later control and the later intervention groups).

All procedures were approved by the Friedrich-Alexander-Universität Erlangen-Nürnberg Ethics Committee (Re.-No. 170_14 B).

### Recruitment

All users of the 34 day-care centers throughout Germany and their caregivers were included in the screening process. All dyads (consisting of a day-care center user and caregiver) that fulfilled the criteria for inclusion were informed about the study and asked to take part in the project. Exclusion criteria for the day-care-center users were: blindness, deafness, lacking a caregiver, lacking the ability to communicate, more than one stroke, severe depression, schizophrenia, an addictive disorder, concrete plans for institutionalization, and attendance at the day-care center of less than once a week. The day-care centers’ documentation contained all medical diagnoses and doctors’ prescriptions known to the informal caregivers. Inclusion criteria were informed consent and an MMSE score of 10 or higher. For persons with an MMSE score of 24 or higher, we required them to also have an MoCA score of 22 or lower. Recruitment strategies are described in detail in [26].

### Instruments

#### Tool under investigation

##### Erlangen Test of Activities of Daily Living in Persons with Mild Dementia or Mild Cognitive Impairment (ETAM) [22]

The ETAM is a feasible (19 min on average), reliable, and valid performance test for IADL capabilities in persons with MCI or mild dementia. Thus, it can be administered to investigate the capacity to accomplish complex activities of daily living relevant to older adults living alone. The development of the ETAM was theoretically driven by the International Classification of Functioning, Disability, and Health (ICF), which was published by the World Health Organization (WHO) in 2001 [29]. The ICF is a classification of health and health-related domains including a list of “Body Functions,” “Body Structures,” “Activity and Participation,” and “Environmental Factors.” Activity can be described as the “execution of a task or action by an individual” (p. 123) and Participation can be described as “involvement in a life situation” (p. 123). The domain “Activity and Participation” consists of nine main categories, five of which are particularly relevant for the independent living of persons with dementia [30]: Communication, Mobility, Self-Care, Domestic Life, and Major Life Areas, especially Economic Life. These main categories are represented by six items in the ETAM (one 6-point item for each main category with the exception of two 3-point items for the main category Domestic Life). The total possible score is 30 points with higher values indicating greater competence in the mastery of IADLs. In a first validation study [22], Cronbach’s alpha was .71, and the inter-rater reliability was .97.

#### Control tools

##### Mini-Mental State Examination (MMSE) [31]

The MMSE is the most frequently used short screening instrument for dementia [32]. It assesses five areas of cognitive function: orientation, registration, attention and calculation, recall, and language. Designed to be a short (5–10 min) pencil-and-paper test that is easy to administer, it is based on a total possible score of 30 points, with higher values indicating greater performance capacity. Scores ≥24 points are considered to be indicative of “normal” cognition (not associated with dementia), whereas scores below this can be indicative of mild dementia (18–23 points), moderate dementia (10–17 points), or severe dementia (0–9 points) [33].

##### Montreal Cognitive Assessment (MoCA) [34]

The MoCA is a measure that is used to screen for MCI. It consists of more difficult items than the MMSE and is thus able to better detect MCI [34–37]. Scores range from 0 to 30 points, with higher scores indicating better cognitive performance. A score of 22 or lower indicates cognitive impairment [35].

##### EuroQol five dimensions questionnaire (EQ-5D) [38]

The EQ-5D is a cognitively simple, brief instrument providing a simple description of a person’s generic health status. It consists of five items covering Mobility, Self-Care, Usual Activities, Pain/Discomfort, and Anxiety/Depression. Each item is rated on a 5-point scale indicating the level of severity with higher scores reflecting more complaints.

##### Nurses’ Observation Scale for Geriatric Patients (NOSGER) [39]

The NOSGER is an observer rating scale covering the impairments that are found most frequently in geriatric patients. It consists of six subscales (Mood, Disturbing Behavior, Social Behavior, Memory, ADLs, and IADLs) that contain five items each. We included the Social Behavior subscale in the present study. Each item is rated on a scale that ranges from 1 (always) to 5 (never) with higher scores indicating less impairment.

#### Other measures

Each participant’s age, gender, nursing care needs, and other sociodemographic data were provided by their caregivers or nurses at the day-care center. In Germany, nursing care needs are determined on the basis of a three-level scale to establish eligibility for nursing care benefits. Three care levels describe the extent to which the patient is eligible to receive assistance from long-term care insurance ranging from mild care (level 1) to moderate care (level 2) to a great need for care (level 3).

#### Classification of the level of cognitive impairment (MCI, mild or moderate dementia)

In order to differentiate between day-care-center users with MCI, mild dementia, or moderate dementia, we administered a combination of the MMSE and the MoCA at baseline. The MoCA was administered when the MMSE values ranged from 24 to 30 points, as the MMSE is widely regarded as not being sensitive enough to be able to detect MCI in the range of non-dementia cases [33, 34, 40]. Freitas [35] suggested using a cut-off score of 22 points to discriminate between normal cognition and MCI for the MoCA. To differentiate between mild and moderate dementia, we used the MMSE values and the recommendations by Tombaugh et al. [33]. We considered scores between 18 and 23 points to be indicative of mild dementia and scores between 10 and 17 points to be indicative of moderate dementia. With this procedure, we defined the level of cognitive impairment in a psychometric way.

### Data recording

The MMSE, MoCA, ETAM, and anamnestic data were recorded by staff at the day-care centers who had attended training sessions. The EQ-5D and the NOSGER subscale Social Behavior were completed via computer-assisted telephone interviews (CATIs) with the caregivers. All of the persons involved in data recording were thoroughly trained in the use of each instrument.

### Data quality management

In order to ensure the validity of the data, the data sources (tests, CATIs, day-care centers) are subjected to a random internal audit. To obtain evidence of the inter-rater reliability of the ETAM test and the CATI, 5% of the baseline data were collected with the participation of a second person who was there to observe. For additional information, please see [28].

### Sample

For the purpose of this study, our analyses were based on 443 day-care-center users with baseline data. The proportion of women in the sample was 61.4%, and the mean age was 81.7 years (SD = 7.7). All analyses except for one were based on these 443 day-care-center users and their baseline data (Table 1). Only for the analysis of sensitivity to change did we use the baseline and follow-up data of 151 control-group participants (because of treatment effects, we excluded the intervention group, see Fig. 1).

**Table 1:** Sample characteristics

Characteristics	*N* = 443
Age, M (SD)	81.7 (7.7)
Women, *n* (%)	272 (61.4%)
Education, *n* (%)	
Not completed	24 (5.4%)
9 years	317 (71.6%)
10 years	51 (11.5%)
13 years	23 (5.2%)
More than 13 years	28 (6.3%)
Care level	
No care level	71 (16%)
Level 1	232 (52.4%)
Level 2	136 (30.7%)
Level 3	4 (0.9%)
MMSE score, M (SD)	19.4 (4.7)
Cognitive Impairment according to MMSE/MoCA	
MCI (MMSE 24–30 and MoCA ≤22)	91 (20.5%)
Mild dementia (MMSE 18–23)	186 (42.0%)
Moderate dementia (MMSE 10–17)	166 (37.5%)
ETAM score at baseline, M (SD)	17.3 (7.2)

*MMSE* mini-mental status examination, *ETAM* Erlangen test of activities of daily living in persons with mild dementia or mild cognitive impairment

### Statistical analysis

#### Reliability and item analysis

In order to determine the test construction characteristics of the ETAM, measures of reliability were computed and an item analysis was conducted. Means and standard deviations were calculated at the item level and for the ETAM total score. Cronbach’s α was computed as a measure of internal consistency, and values higher than 0.7 were considered acceptable [41]. The difficulty index and discrimination power were calculated at the item level. As the ETAM items use a multilevel (4- or 7-step) response format, the difficulty index was calculated as the ratio of the subjects’ squared points to the number of subjects times the squared item maximum ($\frac{\sum_{i=1}^n{x}_i^2}{n\bullet {x}_{max}^2}$) [42]. The item difficulty index ranges from 0 (most difficult item) to 1 (easiest item). Item difficulties in the range of .2 to .8 are preferred [43]. Discrimination power was calculated as the corrected item-total correlation. A discrimination power of .3 to .5 should be rated as moderate, whereas a discrimination power > .5 should be rated as high [43].

In order to assess the extent to which the ETAM could discriminate between different levels of severity of cognitive impairment, we computed a one-way ANOVA with the total ETAM score as the dependent variable and the severity of cognitive impairment (MCI, mild dementia, moderate dementia) as the independent variable. For a post hoc analysis, we computed a Games-Howell test because the groups did not have equal variances. Cohen’s d was used to examine the magnitude of the differences in ETAM scores between the subgroups (MCI, mild dementia, moderate dementia).

#### Confirmatory factor analysis

A confirmatory factor analysis was computed to determine whether the six ETAM items fit the proposed one-factor model as found when the exploratory factor analysis was conducted in the first validation study [22]. The asymptotic distribution free (ADF) method of estimation was chosen because the ETAM items were not normally distributed.

To evaluate the model, we used the adjusted chi-square test statistic in conjunction with other fit indices as recommended by Brown [44]. Schreiber et al. [45] recommended the ratio of χ^2^ to df ≤ 2, a root mean square error of approximation (RMSEA) < .06, a comparative fit index (CFI) ≥ .95, and a Tucker-Lewis index (TLI) ≥ .96.

#### Validity

##### Discriminant validity

To test for discriminant validity, the ETAM score at baseline was correlated with several scales or items that measured different constructs (Spearman Rank Sum Correlation) at baseline. The MMSE was used to measure cognition, the Social Behavior scale of the NOSGER was used to measure social behavior, and the five items of the EQ-5D were used to measure specific topics that are relevant for health status: Mobility, Self-Care, Usual Activities, Pain/Discomfort, and Anxiety/Depression. We hypothesized that the correlation between the ETAM and the MMSE would be around .5 because the two tests measure the progression of the same disease, whereas the correlations between the ETAM and the other tests were expected to be low (.2 or lower).

##### Criterion-related validity

The variable nursing care needs is an appropriate independent external criterion that was used to assess criterion-related validity. It is determined by external raters working for the “Medical Service of Health Insurances” and is a health measure with relevance to a person’s economic standing because it determines the amount of access a person has to financial assistance. We wanted to test the hypothesis that participants achieve significantly different ETAM scores depending on their care level. We computed a one-way ANOVA with the ETAM score at baseline as the dependent variable and nursing care needs as the independent variable. For a post hoc analysis, we computed Hochberg’s GT2 because the population variances were equal but the sample sizes were very different.

##### Sensitivity to change: Subgroup analysis of the control group

We wanted to assess the ETAM’s sensitivity to reflect change in cognitive abilities that occurred over six months in the subgroup of participants in the control group (*n* = 151) from the “DeTaMAKS” project. We expected that participants with larger decreases in their cognitive abilities as measured with the MMSE would also show larger decreases in their IADL capacities as measured with the ETAM. A regression analysis was computed with the ETAM score at follow-up (t1) as the criterion and the ETAM score at baseline (t0) and the MMSE change score from t0 to t1 as predictors.

The analyses were computed on the baseline data for the sample consisting of participants with MCI, mild dementia, or moderate dementia (*N* = 443) and for the subgroups with MCI (MMSE score 24–30 and MoCA ≤22), mild dementia (MMSE score 18–23), and moderate dementia (MMSE score 10–17). For reasons of comparability, we also report results for the original group that was targeted by the ETAM (i.e. persons with MCI or mild dementia; *n* = 277) when similar analyses were carried out in the first validation study [22].

IBM SPSS Statistics 21 was used for most of the statistical analyses. Stata 13.1 was used for the confirmatory factor analysis.

## Results

### Reliability and item analysis

**Fig. 2:**
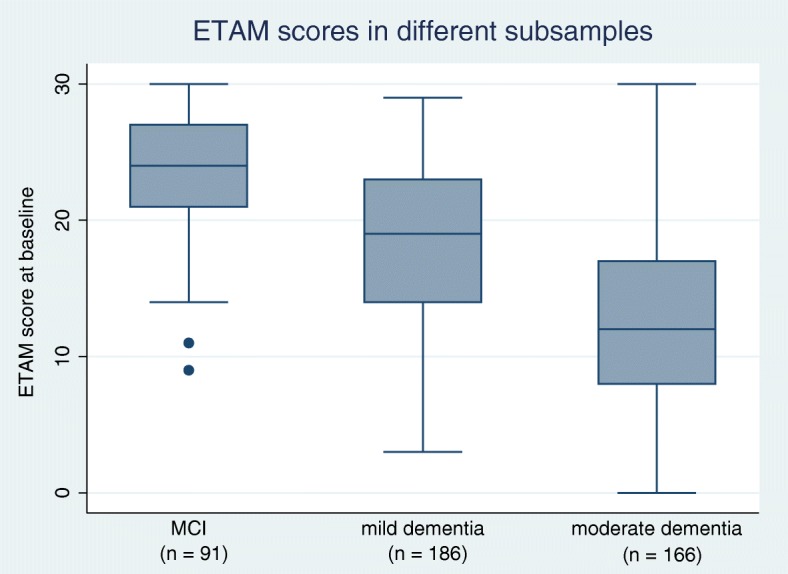
Boxplots of the ETAM scores in persons with MCI, mild dementia, or moderate dementia

For the total sample consisting of participants with MCI, mild dementia, or moderate dementia (*N* = 443), the mean ETAM score was 17.3 points with a standard deviation of 7.2. The median was 18.0 points. The distribution had a skewness of −.264 and a kurtosis of −.852. The maximum range of 0 to 30 points was completely covered. Cronbach’s alpha was .79. For the group comparisons, the following Cohen’s d values were found: d = 0.84 for MCI (*n* = 91) versus mild dementia (*n* = 186), d = 1.70 for MCI versus moderate dementia (*n* = 166), and d = 0.85 for mild versus moderate dementia. For the original target group of participants with MCI or mild dementia (*n* = 277), the mean ETAM score was 20.0 points (SD = 6.2) with a Cronbach’s alpha of .74. The mean ETAM scores differed significantly between MCI, mild dementia, and moderate dementia, F(2, 440) = 87.85, *p* < .001. A post hoc Games-Howell test revealed that all ETAM scores differed significantly from each other at p < .001: Participants with MCI (n = 91) scored on average 23.2 points (95% CI 22.2–24.2), participants with mild dementia (n = 186) scored 18.4 points (95% CI 17.5–19.3), and participants with moderate dementia (*n* = 166) scored 12.9 points (95% CI 11.9–14.0); see Fig. 2.

The overall discriminatory powers were high, ranging from .49 to .64. Only in the subgroup of persons with MCI did the item “alarm clock” have a low discriminatory power of .25. Other items in this subgroup had moderate discriminatory powers ranging from .35 to .44. In the subgroup of persons with mild dementia, the discriminatory power ranged from .45 to .56, and in the subgroup of persons with moderate dementia, from .39 to .63. Overall, the most difficult item was “phone call” (.25), whereas “making tea” (.67) was the easiest item. Again, only for the subgroup of persons with MCI, the easiest items were “pill organizer” and “alarm clock” (both .83) for which the difficulties ranged from .44 to .83. In the subgroup of persons with mild dementia, the difficulties ranged from .25 to .71. In the subgroup of persons with moderate dementia, the difficulties ranged from .13 to .56. Item characteristics are presented in Table 2.

**Table 2:** Item characteristics of the ETAM

Characteristics	Making tea	Alarm clock	Pill organizer	Finances	Traffic situations	Phone call	ETAM total score
Score Range	0–3	0–3	0–6	0–6	0–6	0–6	0–30
Mean (SD)							
Total	2.3 (1.0)	2.2 (0.9)	3.7 (2.3)	3.6 (2.0)	3.3 (1.9)	2.4 (1.8)	17.3 (7.2)
MCI	2.5 (0.8)	2.7 (0.6)	5.3 (1.4)	5.0 (1.5)	4.2 (1.6)	3.6 (1.7)	23.2 (4.7)
Mild dementia	2.4 (0.9)	2.3 (0.9)	4.0 (2.1)	3.7 (1.9)	3.5 (1.7)	2.5 (1.7)	18.4 (6.2)
Moderate dementia	2.0 (1.1)	1.7 (1.0)	2.5 (2.3)	2.7 (2.0)	2.5 (1.9)	1.6 (1.5)	12.9 (6.7)
Discriminatory power							
Total	.49	.53	.59	.61	.54	.64	
MCI	.37	.25	.35	.44	.42	.40	
Mild dementia	.50	.45	.46	.46	.51	.56	
Moderate dementia	.45	.42	.50	.59	.39	.63	
Difficulty							
Total	.67	.61	.53	.47	.39	.25	
MCI	.77	.83	.83	.44	.57	.44	
Mild dementia	.71	.66	.58	.48	.42	.25	
Moderate dementia	.56	.43	.31	.31	.27	.13	
Cronbach’s alpha if item deleted							
Total	.78	.77	.75	.74	.76	.73	
MCI	.60	.62	.59	.55	.56	.57	
Mild dementia	.69	.70	.70	.69	.67	.65	
Moderate dementia	.72	.72	.70	.66	.73	.66	

*N* = 443; n(MCI) = 91; n(mild dementia) = 186; n(moderate dementia) = 166

MCI: MMSE score 24–30; mild dementia: MMSE score 18–23; moderate dementia: MMSE score 10–17

### Confirmatory factor analysis

We hypothesized a one-factor model. For the extended total sample of persons with MCI, mild dementia, or moderate dementia, the model indicated a good fit to the data, χ^2^ (9, *N* = 443), *p* = .088. The ratio of χ^2^ to df was 1.68. The CFI was .975, the TLI was .959, and the RMSEA was .039. Similar results were found for the original target group of participants with MCI or mild dementia: χ^2^ (9, *n* = 277), *p* = .359. The ratio of χ^2^ to df was 1.10. The CFI was .991, the TLI was .985, and the RMSEA was .019. These values indicate a good fit between the model and the observed data.

### Validity

#### Discriminant validity

Overall, the correlation of the ETAM total score with the MMSE was .59; for the subgroups, the correlations were .20 for MCI, .24 for mild dementia, and .40 for moderate dementia. For the original target group of participants with MCI or mild dementia, the correlation with the MMSE was .43. The ETAM total score was hardly correlated with the other items of the EQ-5D: Mobility −.00, Self-Care −.23, Usual Activities −.20, Pain/Discomfort .15, Anxiety/Depression .01. The correlation with the Social Behavior scale from the NOSGER was −.11. The correlations between the ETAM total score and the scores from these instruments are presented in Table 3 in detail.

**Table 3:** Discriminant validity

	Total	MCI	Mild dementia	Moderate dementia
MMSE	.59^**^	.20	.24^**^	.40^**^
Social Behavior (NOSGER)	−.11^*^	.03	−.04	−.01
EQ-5D items				
Mobility	−.01	.12	−.08	−.07
Self-Care	−.23^**^	−.10	−.18^*^	−.18^*^
Usual Activities	−.20^**^	−.14	−.20	−.08
Pain/Discomfort	.15^**^	.17	.05	.05
Anxiety/Depression	.01	.03	.03	−.03

^*^p < .05; ^**^p < .01

#### Criterion-related validity

We computed a one-way ANOVA to test the hypothesis that participants would receive different ETAM scores depending on their care level. The independent variable care level had three factor levels: no care level, care level 1, and care level 2+. We combined participants with care levels 2 and 3 because there were only four participants with care level 3. The results showed that the ETAM scores differed significantly from each other depending on the participants’ nursing care needs, F(2, 440) = 8.660, *p* < .001, as shown in Table 4. Hochberg’s GT2 post hoc test showed that there were significant differences in ETAM scores between participants with no care level and care level 2+ (p < .001) and participants with care level 1 and care level 2+ (*p* = .015). Participants with no care level and care level 1 did not differ significantly in their ETAM scores (*p* = .11).

**Table 4:** Criterion-related validity

	Number	M	SD	p, Hochberg’s GT2 post hoc test	
				Care level 1	Care level 2+
No care level	71	19.70	6.18	.11	< .001
Care level 1	232	17.69	7.08		< .05
Care level 2+	140	15.55	7.57		

#### Sensitivity to change: Subgroup analysis of the control group

In a regression analysis, when predicting the ETAM score at t1, we found that the ETAM score at t0 (b = 0.83, p < .001) and the change in MMSE from t0 to t1 (b = − 0.37, p < .001) were significant predictors; see Table 5. R^2^ was 0.65. This supports our hypothesis that when a person’s MMSE score had declined after six months, the person also achieved fewer points on the ETAM after six months.

**Table 5:** Sensitivity to Change: Subgroup Analysis of the Control Group: Regression Analysis

	Estimate (b)	SE	*p*	95% CI	
				lower limit	upper limit
ETAM score at t0	0.83	0.05	< .001	.721	.937
MMSE change score t0-t1	−0.37	0.10	< .001	−.566	−.181

## Discussion

In this study, we examined the reliability and validity of the ETAM and confirmed that the ETAM can be used not only with people with MCI and mild dementia but also with people with moderate dementia. We showed that ETAM scores differed between the level of cognitive impairment with people with MCI achieving the best results, people with mild dementia second best, and people with moderate dementia the worst. In addition, we confirmed that the ETAM is able to detect change over time. Also, a confirmatory factor analysis supported the postulated single factor structure of IADLs.

The present study supports the application of the ETAM for persons with MCI or mild dementia. In addition, the ETAM can also be recommended for assessing the subgroup of persons with moderate dementia. This is meaningful because functional assessment becomes more important when the degree of cognitive impairment increases [5]. Our analyses showed that persons with MCI achieved the best results, persons with mild dementia scored on average five points lower, and persons with moderate dementia scored another six points lower. Thus, these results show that as the dementing disease progresses, participants find it increasingly difficult to carry out the IADL-oriented tasks of the ETAM, thus providing support for the ETAM’s reliability and validity.

Further support for the validity of the ETAM was provided by care level, which is primarily related to BADL capacities. We found that participants who had not yet qualified for a care level achieved the most points (i.e. they showed a better performance on the ETAM), and with a higher care level, participants achieved fewer points on the ETAM. Persons with no care level and persons with care level 1 did not show significantly different ETAM scores, which might be due to the different sample sizes that were used or the fact that care level is more strongly related to BADL capacities than to IADL capacities. This finding is especially interesting because care level is an external criterion that was rated by independent testers who were not involved in the study.

We were able to confirm the discriminant validity of the ETAM as predicted in our hypotheses (moderate overall correlation with the MMSE; low correlations with all other tests). Whereas the ETAM scores of people with MCI were barely correlated with the MMSE, the correlation increased when we analyzed the subgroup of persons with mild or moderate dementia. This finding is consistent with Giebel et al.’s [46] results in suggesting that with the progression of the dementing disease, cognition is increasingly affected, and people have more trouble mastering IADLs. Further support for the association between cognitive levels and functional abilities such as IADLs was found, for example, by Njegovan [47], who showed that progressive cognitive decline is associated with a specific pattern of loss of functional tasks. All in all, these findings appear to suggest that activities of daily living and cognitive tasks are increasingly associated as cognitive impairment progresses. This means that the relationship between IADL capacities and performance on cognitive tasks increases as cognitive impairment progresses. A similar yet weaker pattern was found for the correlation between the ETAM and the Self-Care item from the EQ-5D, which can be applied to assess BADLs to a certain extent. Again, as the dementing condition progressed, the correlation with the ETAM increased.

In addition, we used the five EQ-5D items to compute correlation coefficients with dimensions such as pain, anxiety, etc. Aside from Self-Care, we found no meaningful correlation or pattern of correlations across the three subgroups of participants with MCI, mild dementia, or moderate dementia, thus providing support for the discriminant validity of the ETAM.

Another important relationship between the ETAM and the MMSE concerns sensitivity to change. For this purpose, we analyzed whether the ETAM was sensitive to other (cognitive) changes over a period of six months. We found that the change in MMSE over a period of six months turned out to be a significant predictor of the ETAM score after six months: When a person’s MMSE score had declined after six months, the person also achieved fewer points on the ETAM after six months. This is an important aspect of validity and it demonstrates that the ETAM is able to measure change over time. Thus, we recommend its use in intervention studies.

Similar to the first validation study, the item “phone call” turned out to be the most difficult item by far. The authors of the first validation study argued that how a person handles the phone is an important and sensitive indicator of incipient dementia processes [22]. The item “traffic situations” was the second most difficult item. Apart from these findings, there were some differences in the order of items in comparison with the first validation study. This was most likely due to a smaller sample size in the previous study as well as less variation (the difficulties of the remaining four items ranged only from .47 to .67). In the current study, there was a consistent pattern of difficulty indices with one small exception. For the MCI subgroup, “pill organizer” and “alarm clock” were the easiest items (both .83), and “making tea” was the third easiest item (.77). Because it is common practice to arrange the items on a test in order of increasing difficulty, we propose that the order of the ETAM items be rearranged and adjusted to reflect the difficulties found in the current study. Specifically, we suggest the following order when carrying out the ETAM: 1) “making tea,” 2) “alarm clock,” 3) “pill organizer,” 4) “finances,” 5) “traffic situations,” 6) “phone call.” When the items are administered in this order, the participant is encouraged to continue the test, and this will also ensure that weaker candidates will not become discouraged.

### Limitations

Some limitations of the current study should be mentioned. Because the lack of high-quality performance-based assessments for measuring IADL capabilities was the reason we developed the ETAM, we cannot provide convergent validity with other instruments that measure IADL capacities. To date, there is no gold standard for measuring IADL capacities especially by means of a performance-based assessment. Existing performance-based assessments are very time-consuming, taking up to 1.5 h [48] in only very small groups [24], or they seem to measure cognition rather than IADL functioning [49] (for an overview, see [22]). Because the ETAM already showed acceptable convergent validity with the informant-based Bayer Activities of Daily Living Scale [50] in the first validation study [22], we decided to focus on discriminant validity and sensitivity to change.

In addition, one should consider that differentiating between MCI, mild dementia, and moderate dementia can be performed only with the mean ETAM scores. This is because there is high between-subject variability on the ETAM in the three levels of cognitive impairment. Thus, ETAM scores should not be used to diagnose MCI, mild dementia, or moderate dementia.

Another limitation of the present study was that we used the NOSGER subscale Social Behavior and the EQ-5D items to analyze discriminant validity. However, a measure of mood would have been desirable because depressive mood is associated with a decline in cognitive abilities.

### Future research perspectives

In our study, the categorization of MCI, mild dementia, and moderate dementia was solely based on the cognitive tests of the MMSE and the MoCA, which can be influenced by age and education [51, 52]. Thereby, we defined cognitive impairment psychometrically and assessed clinical symptoms. In practice, the MMSE is one of the most commonly used screening tools for cognitive impairment [32], and our analyses also showed that this categorization was successful. For a more accurate categorization for persons with MCI and different stages of dementia, future studies could focus on the use of other instruments besides the MMSE as well (e.g. the Consortium to Establish a Registry for Alzheimer’s Disease [CERAD], neuroimaging, and biomarkers). Especially in the preclinical and prodromal stages (MCI) of Alzheimer’s disease, biomarker assessments are very informative [5].

## Conclusions

There is further evidence for the ETAM as a feasible, reliable, and valid instrument for the measurement of IADL capacities in persons with MCI or mild dementia. In addition, the ETAM can be recommended for the assessment of the IADL capacities of persons with moderate dementia. It shows good discriminant validities with other measures (e.g. Social Behavior, Mobility, Pain/Discomfort, and Anxiety/Depression). The ETAM is sensitive to change, and thus, we recommend its use for intervention studies.

## Abbreviations

ADLs: Activities of daily living
BADLs: Basic activities of daily living
CATI: Computer-assisted telephone interview
CFA: Confirmatory factor analysis
CFI: Comparative fit index
CI: Confidence interval
DAFA: Direct assessment of functional abilities
DAFS: Direct assessment of functional status
E-ADL: Erlangen test for activities of daily living
EQ-5D: EuroQol five dimensions questionnaire
ETAM: Erlangen test of activities of daily living in persons with mild dementia or mild cognitive impairment
FLSA: Functional living skills assessment
IADLs: Instrumental activities of daily living
ICF: International classification of functioning, disability, and health
MCI: Mild cognitive impairment
MMSE: Mini-mental status examination
MoCA: Montreal cognitive assessment
NOSGER: Nurses’ observation scale for geriatric patients
RMSEA: Root mean square error of approximation
SD: Standard deviation
TLI: Tucker-Lewis index
WHO: World Health Organization
